# The impact of diagnosis-related group payment on the hospitalization expenditure and medical quality of public hospitals in China

**DOI:** 10.1371/journal.pone.0336527

**Published:** 2025-11-14

**Authors:** Mei Zhou, Yufan Mao, Zizhuo Jiao, Liangrong Zhou

**Affiliations:** School of Humanities and Management, Hunan University of Chinese Medicine, Hunan, China; University of Lisbon, Institute of Social and Political Sciences, PORTUGAL

## Abstract

**Background:**

Healthcare expenditures in China have been rising rapidly in recent years. To reform the medical insurance payment system, China has introduced Diagnosis-Related Groups (DRG) to maintain quality. But does excessive control of hospitalization expenditures affect the quality of care? This study analyzes the implementation of DRG in Chinese public hospitals to examine its impact on both hospitalization expenditures and quality of care.

**Methods:**

Based on data from the Hospital Information Systems (HIS), Electronic Medical Records (EMR), and the DRG management platform in Hunan Province, this study utilized a random sampling method to select hospitalization data. The analysis included 49,192 cases from four public hospitals, encompassing periods before(n = 23,494) and after(n = 25,698) DRG implementation. Additionally, data from two other public hospitals were randomly selected, comprising 7,969 cases before and after the introduction of hospital administrative interventions following DRG implementation (3,862 pre-intervention and 4,107 post-intervention). Statistical analyses comprised descriptive statistics, t-tests, chi-square tests, multiple linear regression, and multivariate logistic regression.

**Results:**

After DRG implementation, the logarithmic mean of total hospitalization expenditures decreased significantly (3.914 ± 0.837 vs. 3.872 ± 1.004), while rates of unplanned readmissions, unplanned reoperations, postoperative complications, and patient complaints within 30 days increased significantly (3.784% vs 4.214%, 0.083% vs 0.166%, 0.207% vs 0.258%, 3.741% vs 5.133%). The proportions of grade IV surgeries and critical patients also decreased (42.602% vs 46.174%, 16.943% vs 18.001%). Adjusted linear regression indicated DRG implementation was negatively associated with the log mean of costs (β = –0.002, 95% CI: – 0.003, – 0.001), a 0.2% reduction. In logistic regression, DRG was not significantly associated with mortality or nosocomial infection, but was positively associated with postoperative complications (OR = 1.16, 95% CI: 1.12, 1.20) and patient complaints (OR = 1.32, 95% CI: 1.01, 1.75).Post-DRG, provincial hospitals had higher values than municipal hospitals in log mean costs, proportion of critical patients, and Grade IV surgeries (3.897 ± 1.024 vs. 3.857 ± 1.012; 19.568% vs. 16.463%; 47.252% vs. 39.371%). Before DRG, provincial (vs. municipal) hospitals showed no association with hospitalization expenditures, critical illness proportion, or mortality, but had 1.19 times the Grade IV surgery proportion (OR = 1.19, 95% CI: 1.08, 2.32). After DRG, these became 1.004, 1.34, and 1.44 times higher, respectively, with no mortality association. After administrative intervention, increases occurred in nosocomial infection, unplanned reoperation, 30-day readmission, patient complaints (5.955% vs. 5.040%; 0.186% vs. 0.000%; 4.065% vs. 3.324%; 4.868% vs. 3.360%), and log mean costs (3.898 ± 1.253 vs. 3.963 ± 0.884). Mortality and postoperative complications did not change significantly (0.40% vs. 0.463%; 0.279% vs. 0.189%). Linear regression indicated a positive association between intervention and expenditures (0.5%increase). Logistic regression showed negative associate with mortality, infection, complications and patient complaints corresponding to risk reductions of 2%, 7%, 4% and 3% respectively.

**Conclusions:**

The DRG payment system effectively controlled the growth of hospitalization expenditures in Chinese public hospitals. However, an exclusive focus on expenditure containment may adversely affect medical quality. Appropriate administrative interventions can help improve medical quality while managing expenditures.

## Introduction

In recent years, the total health expenditures in China have witnessed a rapid escalation [1]. From 2003 to 2023, the total health expenditure rose from CNY 658.4 billion ($93.1 billion) to CNY 9057.6 billion ($1281.6 billion), marking a more than twelvefold increase. Meanwhile, the per capita medical expenditure skyrocketed from CNY 509.5 in 2003 to CNY 6,425.3 in 2023, with personal healthcare expenditures currently accounting for 27.3% of China’s total healthcare expenditure [2,3]. The escalating medical expenditure has imposed a heavy burden on both China’s healthcare insurance funds and individual patients. For a long time, China’s healthcare insurance payments have primarily followed a fee-for-service (FFS) model. However, some researchers argue that this payment method is a key factor driving the excessive growth of total healthcare costs [4]. Studies have shown that the FFS model, which can lead to a lack of cost-control awareness among healthcare providers, contributes to escalating healthcare costs and the inefficient use of healthcare resources [5]. Additionally, experiences from other countries suggest that adopting a global budget or diagnosis-related group (DRG)-based payment system can help curb the growth of healthcare costs [6]. Empirical research has indicated that the FFS model incentivizes healthcare providers to increase unnecessary medical services and encourage more frequent patient visits, ultimately leading to overutilization. This not only further exacerbates the financial burden on both insurance funds and patients but also results in the inefficient allocation of healthcare resources [5–8]. Therefore, reforming the healthcare insurance payment system to enhance service efficiency is considered one of the most effective measures for controlling the growth of healthcare costs [9].

The Diagnosis-Related Groups (DRG) payment system is a case-based framework that categorizes patients into groups with similar clinical profiles and resource needs to standardize reimbursement rates [10]. Initially developed in the United States, DRG has been widely adopted globally to promote the rational allocation of healthcare resources and improve the efficiency and quality of care. Extensive research demonstrates its successful implementation across various healthcare systems. For instance, in the United States, the introduction of DRG was associated with significantly reduction in hospital length of stay and enhanced institutional efficiencies [11]. In Germany, its implementation contributed to lower medical costs and optimize hospital resource allocation [12]. In South Korea, studies indicate that the DRG system effectively reduced hospitalization expenses while concurrently exerting a positive influence on medical quality [13]. These international experiences offer valuable insights for China’s ongoing DRG payment reforms. China represents a notable case as a developing country actively developing and integrating a localized DRG system into its healthcare financing structure [14].

The implementation of the DRG payment system in China is motivated by two primary objectives. First, it addresses systemic challenges, including the uneven distribution of healthcare resources, rapidly escalating expenditures, and inefficiencies in service delivery. Unlike the traditional fee-for-service model, which has proven inadequate, the DRG system establishes standardized classification and incentives to guide medical institutions toward optimized resource allocation, improved operational efficiency, and controlled growth of healthcare expenditures [15]. Evidence suggests that DRG not only contains costs but also enhances resource utilization efficiency and promotes better hospitals management [16]. However, scholars have noted potential drawbacks, such as incentives for hospitals to preferentially admit low-risk patients while avoiding complex cases, and concerns that premature discharge rates may increase, potentially compromising care quality [17–18]. The second objective of the DRG system is to encourage healthcare institutions to balance cost control with enhancements in service quality and patient safety. Ultimately, by fostering a patient-centered approach, the DRG reforms aim to steer China’s healthcare system toward high-quality development [19].

However, the implementation of the DRG payment system is not without challenges and risks. A central challenge lies in balancing cost containment with the maintenance of medical quality and patient safety. Comprehensive quality assessment typically encompasses multiple dimensions, such as clinical outcomes, patient safety, service efficiency, and patient satisfaction. Common metrics include 30-day unplanned readmission rates, postoperative complication rates, unplanned reoperation rates, inpatient mortality, and patient complaints or satisfaction surveys. These multidimensional metrics facilitate a holistic evaluation of care quality and support service optimization [20]. Research suggests that an overemphasis on cost reduction may incentivize hospitals to deviate from clinical best medical practices in treatment and discharge planning, potentially compromising patient outcomes and safety [21–24]. Specifically, the DRG system has been associated with shorter lengths of stay, which may increase readmission rates, particularly following surgical procedures and in chronic disease management [18,25]. Furthermore, some institutions might respond to financial pressures by reducing high-cost interventions or diluting service standards, adversely affecting clinical outcomes [26]. Consequently, the successful implementation of DRG reforms necessitates carefully designed incentive structures and robust regulatory oversight to ensure that quality and safety are safeguarded.

This study investigates the impact of the Diagnosis-Related Groups(DRG) payment system on hospitalization expenditures and medical quality within Chinese public tertiary hospitals, a key focus is placed on examining regional variations in its implementation. Furthermore, the research assesses the role of hospital-level administrative interventions in moderating these outcomes following DRG adoption. The findings are expected to offer valuable theoretical insights and empirical evidence to inform the ongoing optimization of healthcare payment reform policies in China.

## Methods

### Data source and sampling

This study analyzed data from the Hospital Information System (HIS), the Electronic Medical Record (EMR) system, and the DRG management platform. To evaluate the impact of DRG implementation, we employed a stratified random sampling method to select cases from four Tertiary Grade A hospitals. Specifically, we selected a monthly sample of 8.33% of all hospitalized patients from January to December 2022 (post-DRG) and, for a pre-DRG control group, from the same period in 2019 (January-December), yielding initial samples of approximately 30,000 and 25,000 cases, respectively. To assess the effect of administrative interventions, we similarly sampled 10,000 cases from two other Tertiary Grade A public hospitals in 2021.

### Study population

**Fig 1:**
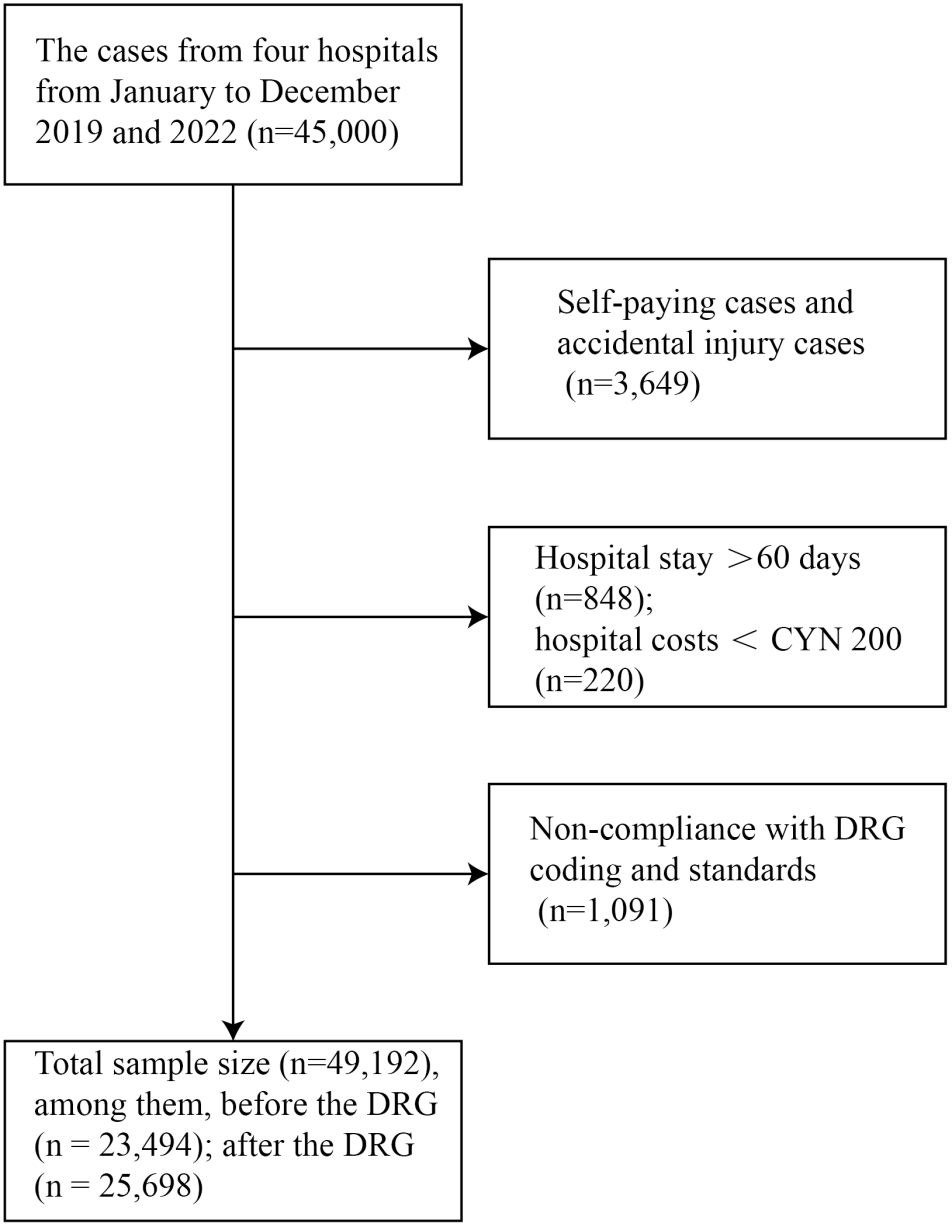
Flowchart of case sample selection before and after implementation of DRG in four hospitals in 2019 and 2022.

**Fig 2:**
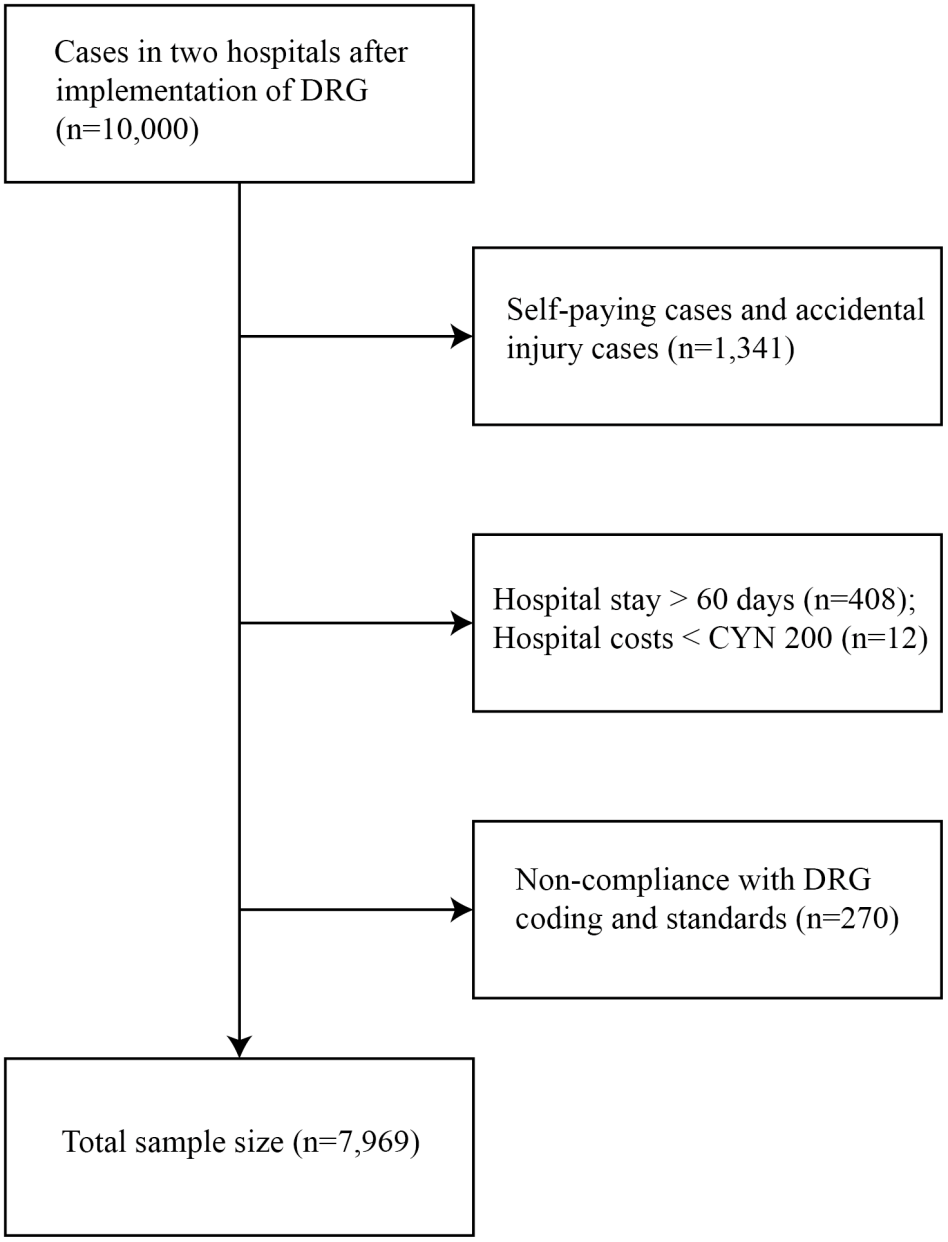
Flowchart of case sample selection for hospital administrative management intervention after DRG implementation in two hospitals in 2021.

The following cases were excluded from all cohorts: self-paying patients, those with a hospital stay >60 days, hospitalization expenditures < CNY 200, and cases not meeting DRG coding criteria. After exclusions, the final sample for the DRG implementation analysis consisted of 25,698 post-DRG cases (2022) and 23,494 pre-DRG control cases (2019). The sample for the administrative intervention analysis included 7,969 cases (3,862 pre-intervention and 4,107 post-intervention). The distribution of cases between provincial and municipal hospitals is summarized in the results. The selection process is illustrated in Figs 1 and 2.

### Data processing and quality control

All sensitive patient information was de-identified to ensure privacy. The data was processed through a robust cleaning pipeline, which included the evaluation and appropriate handling of clinically meaningful outliers, the removal of illogical or erroneous entries, and the use of multiple imputation to address missing data. Multiple rounds of validation were conducted by the research team to guarantee data accuracy and consistency.

#### Assessment of medical quality.

Medical quality was assessed using the following outcome indicators [27]:

**Nosocomial infection:** infections occurring >48 hours after admission, involving the lungs, urinary tract, bloodstream, or surgical sites [28].

**Hospital mortality:** the proportion of in-hospital deaths among all admitted patients over a specific period [29].

**30-day unplanned readmission:** a hospital readmission within 30 days of discharge due to unforeseen reason [30].

**Unplanned reoperation:** a subsequent surgery required due to complications or other unanticipated issues following an initial procedure [31].

**Postoperative complications:** adverse events occurring after surgery, such as infections, bleeding, or organ dysfunction [32].

**Patient complaint:** formal or informal expression of dissatisfaction regarding healthcare services from patients or their families [33].

#### Variables.

Based on existing literature, potential confounders affecting medical quality were assessed [34].

#### Sociodemographic and Lifestyle Variables.

**Age** was categorized into three groups: ≤ 14, 15–60, and >60 years.

**Gender** was recorded as male or female.

**Education level** was classified as high school or lower, junior college, undergraduate, master’s, or doctoral.

**Marital status** was grouped as married/cohabiting, widowed/divorced/ separated, or never married.

**Smoking status** (yes/no) was defined as smoking at least 4–5 cigarettes daily [35].

**Alcohol use** (yes/no) was defined as consuming at least 10 units of alcohol per month, where one unit equated to 45 mL of spirits, 350 mL of beer, or 150 mL of wine [36].

#### Clinical Variables.

**Hypertension** (yes/no) was defined by at least one of the following: systolic/diastolic blood pressure ≥140/90 mmHg, a prior physician diagnosis, or current use of antihypertensive medication [37].

**Diabetes** (yes/no) was defined by at least one of the following: fasting blood glucose ≥7.0 mmol/L, glycated hemoglobin ≥6.5%, a physician diagnosis, or current use of insulin/glucose-lowering drugs [38].

**Cardiovascular disease** (yes/no) was defined as a physician-diagnosed condition or current use of cardiovascular medications [39].

**Antibiotic utilization** (yes/no) was recorded if any antibiotics were administered during the hospitalization.

**Grade IV operations** (yes/no) referred to major, complex surgical procedures performed after admission [40].

**Acute and critical cases** (yes/no) referred to patients admitted through emergency departments with abnormal vital signs [40].

### Statistical analysis

Descriptive results are presented as mean ± standard deviation (SD) for continuous variables, and as frequencies with percentages for categorical variables. In regression analyses, odds ratio (OR) and 95% confidence interval (CI) are reported. Given the strong right-skewness in hospitalization expenditure data, a base-10 logarithmic was applied prior to analysis. Continuous variables were compared using independent samples t-tests, and categorical variables were compared using Rao–Scott chi-square tests.

First, to assess whether DRG payment impacts medical quality and safety, we compared indicators including nosocomial infection rate, mortality rate, 30-day unplanned readmission rate, antibiotic usage rate, proportion of grade Ⅳ surgeries, proportion of acute and critical patients, unplanned reoperation rate, and postoperative complication rate before and after DRG implementation. Second, to explore potential transfer of acute, critical, and complex patients from municipal hospitals to provincial hospitals after DRG implementation, we compared average total hospitalization expenditures, proportion of acute and critical patients, proportion of grade IV surgeries, and mortality rates between municipal and provincial hospitals before and after DRG.

Third, to evaluate the impact of hospital administrative intervention on hospitalization expenditures and medical quality after DRG implementation, a comparative analysis was conducted on the following indicators before and after the intervention in two additional public hospitals: the logarithmic mean of total hospitalization expenditures, nosocomial infection rate, unplanned reoperation rate, in-hospital mortality rate, 30-day readmission rate, postoperative complication rate, and patient complaint rate. (It should be noted that hospital administrative intervention refers to incentive measures based on case quality implemented by attending physicians within their jurisdiction.)

Finally, to determine whether the observed changes in the logarithmic mean of total hospitalization expenditures and medical quality indicators following DRG implementation and administrative intervention were independent of potential confounding factors, we performed multivariable analyses. Multiple linear regression was used for log-transformed expenditures, and multiple logistic regression was used for binary quality outcomes (nosocomial infection, in-hospital mortality, postoperative complications, and patient complaints). The adjustment strategies for the regression models were as follows:

Model 1: Unadjusted.

Model 2: Adjusted for age and gender only.

Model 3: Fully adjusted for all covariates, including age, gender, education level, marital status, alcohol use, smoking status, hypertension, diabetes, cardiovascular disease, antibiotic use, Grade IV surgery status, and acute/critical case status.

All statistical analyses were performed using SPSS (version 24.0) and R (version 4.3.1, R Development Core Team). A two-side p-value < 0.05 was considered statistically significant.

**Fig 3:**
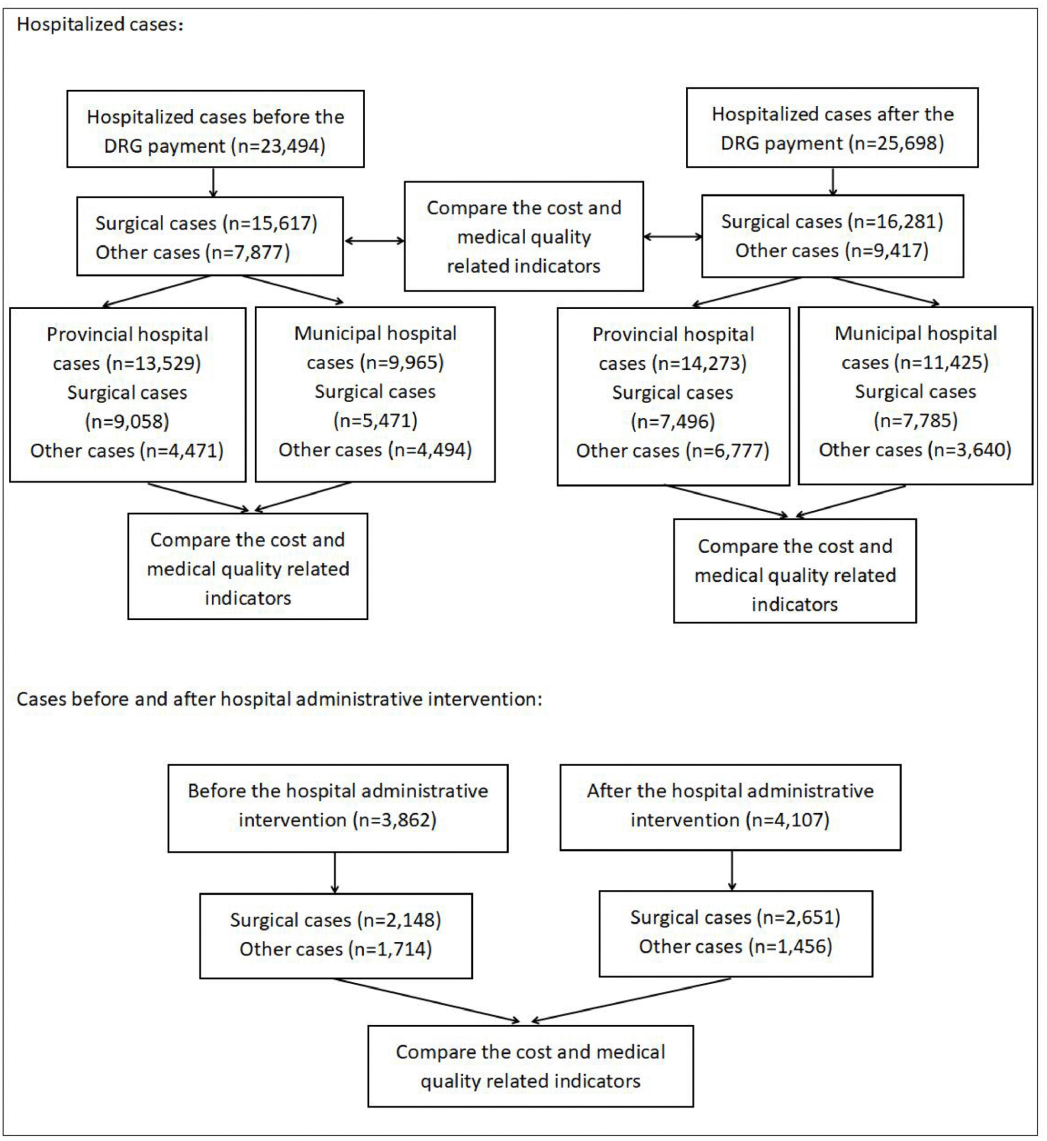
The framework diagram of this study.

The framework diagram of the article is shown in Fig 3.

## Results

### The baseline characteristics of participants before and after DRG Payment

A total of 49,192 participants were included in this study, and their baseline characteristics are shown in Table 1. The cohort was stratified into two groups based on the timing of DRG payment implementation: the pre-DRG group and the post-DRG group. Except for gender and marital status, significant differences were observed between the two groups in terms of age distribution, educational attainment, drinking and smoking status, as well as the prevalence of hypertension, diabetes, and cardiovascular disease (all *p* < 0.05).

**Table 1:** Baseline characteristics of participants.

Variables	Before the DRG Payment (n = 23,494)	After the DRG Payment (n = 25,698)	P value
Age n (%)			<0.001
≤14	3,651 (15.54%)	3873 (15.07%)	
15-60	12,140 (51.68%)	14897 (57.97%)	
≥60	7,703 (32.78%)	6928 (26.96%)	
Gender n (%)			0.064
Male	13,548 (57.67%)	14607 (56.84%)	
Female	9,946 (42.33%)	11091 (43.16%)	
Educational level n (%)			<0.001
High school or lower	11,640 (49.55%)	12609 (49.07%)	
Junior college	6,983 (29.72%)	7271 (28.29%)	
Undergraduate college	4,305 (18.32%)	5120 (19.92%)	
Master	462 (1.97%)	524 (2.04%)	
Doctor	104 (0.44%)	174 (0.68%)	
Marital status n (%)			0.070
Married or Living with Partner	19,151 (81.51%)	21056 (81.93%)	
Widowed, Divorced or Separated	1,870 (7.96%)	2094 (8.15%)	
Never married	2,473 (10.53%)	2548 (9.92%)	
Smoking n (%)			<0.001
Yes	5,172 (22.01%)	6437 (25.05%)	
No	18,322 (77.99%)	19261 (74.95%)	
Alcohol n (%)			<0.001
Yes	4,664 (19.85%)	5703 (22.19%)	
No	18,830 (80.15%)	19995 (77.81%)	
Hypertension n (%)			<0.001
Yes	7,529 (32.05%)	6428 (25.01%)	
No	15,965 (67.95%)	19270 (74.99%)	
Diabetes n (%)			<0.001
Yes	4,111 (17.50%)	5514 (21.46%)	
No	19,383 (82.50%)	20184 (78.54%)	
Cardiovascular disease n (%)			<0.001
Yes	6,733 (28.66%)	6295 (24.50%)	
No	16,761 (71.34%)	19403 (75.50%)	

% for Categorical variables: p-value was calculated by the chi-square test.

### Transformations of total hospitalization expenditures and related indicators of medical quality before and after DRG Payment

Following the implementation of DRG payment, changes were observed in **total hospitalization expenditures and** related medical indicators. The logarithmic value of total hospitalization expenditures decreased significantly after DRG implementation (3.914 ± 0.837 vs 3.872 ± 1.004; *P *< 0.05). The rates of unplanned readmission (3.784% vs 4.214%), unplanned reoperation(0.083% vs 0.166%), postoperative complications(0.207% vs 0.258%), and patient complaints within 30 days(3.741% vs 5.133%) were significantly higher after DRG implementation compared to the pre-DRG period (all *P *< 0.05); In contrast, the proportions of grade IV operations(46.174% vs 42.602%) and acute and critical patients (8.001% vs 16.943%) significantly decreased (P < 0.05). No significant differences were found in the rates of nosocomial infection (5.916% vs. 6.128%), mortality (0.541% vs. 0.428%), or antibiotic use (48.323% vs. 47.463%) (all *P* > 0.05), as summarized in Table 2.

**Table 2:** Transformations of total hospitalization expenditure and related indicators of medical quality before and after DRG Payment.

Variables	Before the DRG payment	After the DRG payment	T/X^2^ value	P value
Log the total hospitalization expenditure, average	3.914 ± 0.837	3.872 ± 1.004	−5.014	*<*0.001
Nosocomial infection rate n (%)	1,390 (5.916%)	1,575 (6.128%)	0.978	0.323
Mortality n (%)	0.541% (0.541%)	110 (0.428%)	3.241	0.072
30-day unplanned readmission rate n (%)	889 (3.784%)	1,083 (4.214%)	5.908	0.015
Antibiotic utilization rate n (%)	11,353 (48.323%)	12,197 (47.463%)	3.639	0.057
The proportion of grade IV operations n (%)	7,211 (46.174%)	6,936 (42.602%)	41.212	<0.001
The proportion of acute and critical patients n (%)	4,229 (18.001%)	4,354 (16.943%)	9.527	0.002
Unplanned reoperation rate n (%)	13 (0.083%)	27 (0.166%)	4.341	0.037
Incidence of postoperative complications n (%)	324 (0.207%)	431 (0.258%)	10.907	0.001
Patient complaint rate n (%)	879 (3.741%)	1,319 (5.133%)	55.657	<0.001

Mean ± SD for continuous variables: p-value was calculated by T-test.

% for Categorical variables: p value was calculated by chi-square test.

Multiple linear regression analysis, after adjusting for all covariates, indicated that the implementation of DRG was associated with a decrease in the logarithmic mean of total hospitalization expenditures (**β** = −0.002, 95% CI:-0.001, −0.006), corresponding to a 0.2% reduction. According to multiple logistic regression, no significant correlation was observed between hospital mortality and DRG implementation, even after full adjustment (model 3: OR 1.08, 95% CI 0.90–1.29). While an initial positive association was found between DRG implementation and nosocomial infection in the unadjusted model, it became non-significant after adjusting for age and sex (OR = 1.32, 95% CI: 0.99–1.76) and after full adjustment (OR = 1.23, 95% CI: 0.93–1.63). In contrast, a positive association with postoperative complications persisted after full adjustment (OR = 1.16, 95% CI: 1.12–1.20), indicating a 16% increase in the odds of complications following DRG implementation. Similarly, the odds of patient complaints were significantly higher after DRG implementation (OR = 1.32, 95% CI: 1.01–1.75). All results are presented in Table 3.

**Table 3:** Multiple linear regression and multiple logistic regression were used to analyze the relationship between DRG and the logarithmic mean of total hospitalization expenditures and medical quality.

Log the total hospitalization expenditure, average	β (95%CI), p-value		
	Model 1	Model 2	Model 3
Implementation of DRG			
No	Reference	Reference	Reference
Yes	−0.004 (−0.005, −0.001), < 0.001	−0.002 (−0.005, 0.001), < 0.001	−0.002 (−0.006, −0.001),0.0028
**Mortality**	OR (95%CI), p-value		
	**Model 1**	**Model 2**	**Model 3**
Implementation of DRG			
No	Reference	Reference	Reference
Yes	1.30 (0.98, 1.72),0.0680	1.11 (0.99, 1.26),0.0815	1.08 (0.90, 1.29),0.4216
Nosocomial infection	OR (95%CI), *p* value		
	**Model 1**	**Model 2**	**Model 3**
Implementation of DRG			
No	Reference	Reference	Reference
Yes	1.31 (1.04,1.68), 0.024	1.32(0.99,1.76), 0.057	1.23 (0.93,1.63), 0.143
Postoperative complications	OR (95%CI), *p-value*		
	**Model 1**	**Model 2**	**Model 3**
Implementation of DRG			
No	Reference	Reference	Reference
Yes	1.23 (1.19,1.26), <0.0001	1.20 (1.16,1.24), <0.0001	1.16 (1.12,1.20), <0.0001
Patient complaint	OR (95%CI), *p-value*		
	**Model 1**	**Model 2**	**Model 3**
Implementation of DRG			
No	Reference	Reference	Reference
Yes	1.56 (1.23,1.97), < 0.001	1.34 (1.01,1.77), 0.042	1.32 (1.01,1.75), 0.045

Model 1: no covariates were adjusted.

Model 2: age and gender were adjusted.

Model 3: age, gender, educational level, marital status, hypertension, Diabetes, cardiovascular disease, alcohol, smoking, antibiotic utilization, condition of grade IV operations, and acute and critical cases were adjusted.

### Hospitalization expenditure and medical indicators in various regions before and after the implementation of DRG payment

To assess potential regional disparities in the impact of DRG payment, we compared hospitalization expenditure and medical indicators between provincial and municipal hospitals. Before DRG implementation, provincial hospitals had a significantly higher proportion of Grade IV surgeries than municipal hospitals (47.582% vs 45.201%, *P *< 0.05). No significant differences were observed in average total hospitalization expenditures, the proportion of acute/critical patients, or mortality (*P* > 0.05). Following DRG implementation, provincial hospitals demonstrated significantly higher average total hospitalization expenditures (3.897 ± 1.024 vs. 3.857 ± 1.012), a greater proportion of acute/critical patients (19.568% vs. 16.463%), and a higher rate of Grade IV operations (47.252% vs. 39.371%) compared to municipal hospitals (all *P* < 0.05). Mortality rates remained comparable between the two groups (*P* > 0.05), as detailed in Table 4.

**Table 4:** Alterations of hospitalization expenditures and medical indicators among different regions before and after DRG payment.

	Before the DRG payment		*t/X*^*2*^ value	*P* value	After the DRG payment		*t/X*^*2*^ value	*P* value
Variables	Municipal hospital	Provincial hospital			Municipal hospital	Provincial hospital		
Log total hospitalization expenditure, average	3.869 ± 0.978	3.843 ± 1.084	−1.893	0.058	3.857 ± 1.012	3.897 ± 1.024	2.974	0.003
The proportion of acute and critical patients n (%)	176 (17.661%)	256 (18.992%)	0.505	0.477	1,881 (16.463%)	2,793 (19.568%)	41.099	0.000
The proportion of grade IV operations n (%)	2,473 (45.201%)	4,310 (47.582%)	7.763	0.005	3,065 (39.371%)	3,542 (47.252%)	96.645	0.000
Mortality n (%)	58 (0.582%)	80 (0.591%)	0.008	0.927	48 (0.420%)	86 (0.603%)	4.070	0.044

Mean ± SD for continuous variables: p-value was calculated by T-test.

% for Categorical variables: p value was calculated by chi-square test.

Multivariable linear and logistic regression analyses, adjusted for all covariates, were used to compare provincial-level hospitals with municipal-level hospitals (reference) before and after DRG implementation. As shown in Table 5, before DRG implementation, provincial hospitals showed no significant association with the mean logarithm of total hospitalization expenditures, the proportion of critically ill patients, or in-hospital mortality. However, the proportion of Grade IV surgeries in provincial hospitals was 1.19 times that of municipal hospitals (OR = 1.19, 95% CI: 1.08–2.32). After DRG implementation (Table 6), the mean logarithm of total hospitalization expenditures, the proportion of critically ill patients, and the proportion of Grade IV surgeries in provincial hospitals were 1.004 times, 1.34 times, and 1.44 times those of municipal hospitals, respectively. No significant association was found with in-hospital mortality.

**Table 5:** Multivariate linear regression and multivariate logistic regression were used to analyze the changes in average hospitalization expenditures and medical quality between provincial hospitals and municipal hospitals before the implementation of DRG.

Log total hospitalization expenditure, average	β (95%CI), p-value		
	Model 1	Model 2	Model 3
Municipal hospital	Reference	Reference	Reference
Provincial hospital	0.002 (−0.002, 0.007),0.258	−0.002 (−0.009, 0.004),0.443	−0.003 (−0.010, 0.003),0.335
**The proportion of acute and critical patients n (%)**	**OR (95%CI), *p* value**		
	**Model 1**	**Model 2**	**Model 3**
Municipal hospital	Reference	Reference	Reference
Provincial hospital	1.64 (0.83,3.22), 0.374	1.41(0.71,2.79), 0.157	1.19 (0.89,1.31), 0.203
**The proportion of grade IV operations** **n (%)**	**OR (95%CI), *p-value***		
	**Model 1**	**Model 2**	**Model 3**
Municipal hospital	Reference	Reference	Reference
Provincial hospital	1.26 (0.96,1.57), 0.061	1.24 (0.93,1.46), 0.089	1.19 (1.08,2.32), < 0.001
**Mortality n (%)**	**OR (95%CI), *p-value***		
	**Model 1**	**Model 2**	**Model 3**
Municipal hospital	Reference	Reference	Reference
Provincial hospital	1.42 (0.84,2.14), 0.675	1.20 (0.84,2.21), 0.842	1.23 (0.92,1.99), 0.117

Model 1: no covariates were adjusted.

Model 2: age and gender were adjusted.

Model 3: age, gender, educational level, marital status, hypertension, Diabetes, cardiovascular disease, alcohol, smoking, antibiotic utilization were adjusted.

**Table 6:** Multivariate linear regression and multivariate logistic regression were used to analyze the changes in average hospitalization expenditures and medical quality between provincial hospitals and municipal hospitals after the implementation of DRG.

Log total hospitalization expenditure, average	β (95%CI), p-value		
	Model 1	Model 2	Model 3
Municipal hospital	Reference	Reference	Reference
Provincial hospital	0.006(0.001, 0.010),0.011	0.005 (0.000, 0.009),0.030	0.004 (0.001, 0.009),0.041
**The proportion of acute and critical patients n (%)**	**OR (95%CI), *p* value**		
	**Model 1**	**Model 2**	**Model 3**
Municipal hospital	Reference	Reference	Reference
Provincial hospital	1.99 (1.82,2.17), < 0.001	1.67(1.18,2.34), 0.0033	1.34 (1.20,1.50), < 0.001
**The proportion of grade IV operations** **n (%)**	**OR (95%CI), *p-value***		
	**Model 1**	**Model 2**	**Model 3**
Municipal hospital	Reference	Reference	Reference
Provincial hospital	1.65 (1.19,2.26), <0.001	1.73 (1.16,2.60), <0.001	1.44 (1.29,3.04), <0.001
**Mortality n (%)**	**OR (95%CI), *p-value***		
	**Model 1**	**Model 2**	**Model 3**
Municipal hospital	Reference	Reference	Reference
Provincial hospital	2.65 (2.26,3.10), < 0.001	1.36 (1.03,1.51), 0.037	1.30 (0.98,1.46), 0.821

Model 1: no covariates were adjusted.

Model 2: age and gender were adjusted.

Model 3: age, gender, educational level, marital status, hypertension, Diabetes, cardiovascular disease, alcohol, smoking, antibiotic utilization were adjusted.

### Changes in hospitalization expenditure and medical quality under medical administrative intervention after the implementation of DRG payment

To evaluate the impact of internal hospital incentive and penalty measures on medical expenditures and quality, we analyzed data from two other tertiary public hospitals before and after the implementation of these administrative interventions.

The analysis revealed that after the intervention, there were significant reductions in the rates of nosocomial infection (5.955% vs. 5.040%), unplanned reoperation (0.186% vs. 0.000%), 30-day unplanned readmission (4.065% vs. 3.324%), and patient complaints (4.868% vs. 3.360%) (all *P *< 0.05). Conversely, the mean logarithm of total hospitalization expenditures was significantly higher than before the intervention (3.898 ± 1.253 vs. 3.963 ± 0.884, P < 0.05). In contrast, no significant changes were observed in hospital mortality (0.440% vs. 0.463%) or postoperative complication rates (0.279% vs. 0.189%) (P > 0.05), as detailed in Table 7.

**Table 7:** Changes in hospitalization expenditures and Medical Quality under Medical Administrative Intervention upon the Implementation of DRG Payment.

Variables	Before the hospital administrative intervention	After the hospital administrative intervention	t/X^2^ value	P value
Log average total hospital expenditure, average	3.898 ± 1.253	3.963 ± 0.884	2.688	0.007
Nosocomial infection rate n (%)	299 (5.955%)	207 (5.040%)	24.432	<0.001
Unplanned reoperation rate n (%)	4 (0.186%)	0 (0.000%)	6.167	0.013
Mortality n (%)	17 (0.440%)	19 (0.463%)	0.022	0.881
30-day re-admission rate n (%)	157 (4.065%)	133 (3.324%)	3.880	0.048
postoperative complications n (%)	6 (0.279%)	4 (0.189%)	0.430	0.511
Patient complaint rate n (%)	188 (4.868%)	138 (3.360%)	11.532	0.001

Mean ± SD for continuous variables: p-value was calculated by T-test.

% for Categorical variables: p value was calculated by chi-square test.

Multiple linear regression analyses demonstrated that the hospital administrative intervention was positively associated with the logarithmic mean of total hospitalization expenditures in the unadjusted model (Model) 1, the partially adjusted model (Model 2), and the fully adjusted model (Model 3). After adjusting for all variates, the intervention was associated with a 0.5% increase in the logarithmic mean of total hospitalization expenditures. Similarly, multiple logistic regression analyses indicated that the intervention was consistently negatively associated with hospital mortality, nosocomial infection, postoperative complications, and patient complaints across all models. Specifically, after full adjustment for covariates, the intervention was associated with reductions of 2% in hospital mortality, 7% in nosocomial infection, 4% in postoperative complications, and 3% in patient complaints, as summarized in Table 8.

**Table 8:** Multiple linear regression and multiple logistic regression were used to analyze the changes in average hospitalization expenditures and medical quality before and after hospital administrative intervention after the implementation of DRG.

Log average total hospitalization expenditure, average	β (95%CI), p-value		
	Model 1	Model 2	Model 3
Medical administrative Intervention			
No	Reference	Reference	Reference
Yes	0.011 (0.004,0.018), 0.003	0.003 (0.001,0.008), 0.012	0.005 (0.001,0.009), 0.026
**Mortality**	**OR (95%CI), p-value**		
	**Model 1**	**Model 2**	**Model 3**
Medical Administrative Intervention			
No	Reference	Reference	Reference
Yes	0.92 (0.90,0.94), < 0.001	0.94 (0.91,0.96), < 0.001	0.98 (0.95,0.99), < 0.01
Nosocomial infection	OR (95%CI), p value		
	Model 1	Model 2	Model 3
Medical Administrative Intervention			
No	Reference	Reference	Reference
Yes	0.87 (0.65,0.92), 0.004	0.89 (0.68,0.90), 0.011	0.93 (0.78,0.94), 0.026
Postoperative complications	OR (95%CI), p-value		
	Model 1	Model 2	Model 3
Medical Administrative Intervention			
No	Reference	Reference	Reference
Yes	0.91 (0.90,0.95), < 0.001	0.93 (0.92,0.98), < 0.001	0.96(0.93,0.99), < 0.001
Patient complaints	OR (95%CI), p-value		
	Model 1	Model 2	Model 3
Medical Administrative Intervention			
No	Reference	Reference	Reference
Yes	0.92 (0.91,0.94), < 0.001	0.94 (0.93,0.96), < 0.001	0.97(0.94,0.98), < 0.001

Model 1: no covariates were adjusted.

Model 2: age and gender were adjusted.

Model 3: age, gender, educational level, marital status, hypertension, Diabetes, cardiovascular disease, alcohol, smoking, antibiotic utilization, condition of grade IV operations, and acute and critical cases were adjusted.

## Discussion

Under the traditional fee-for-service payment model, hospitals inevitably expanded service volume to increase medical revenue [24]. **In this study, we observe** a reduction in the logarithmic mean of total hospitalization expenditures after the implementation of Diagnosis-Related Groups (DRG). This “fixed - price” payment mechanism has helped curb the irrational growth of medical expenses to some extent [41,42]. While the DRG-driven decrease in hospitalization expenditures is a positive outcome, we also identified a concurrent decline in medical quality, which is noteworthy. Compared to the pre-DRG period, there were increases in the 30-day unplanned readmission rate, unplanned re-operation rate, postoperative complication rate, and patient complaint rate. In logistic regression analyses, after adjusting for covariates including age, gender, education level, marital status, hypertension, diabetes mellitus, heart disease, alcohol consumption, smoking, antibiotic use, grade IV surgery, and critical illness cases, DRG implementation remained independently associated with higher risks of postoperative complications (OR = 1.16, 95% CI: 1.12–1.20) and patient complaints (OR = 1.32, 95% CI: 1.01–1.75). This indicates that after DRG implementation, the risk of postoperative complications and patient complaints increased by 16% and 32%, respectively. A potential reason is that hospitals may have cut diagnosis costs to minimize costs, thereby increasing the risks of misdiagnosis or missed diagnosis. The DRG payment reform, based on the principle of “rewards cost savings and shared responsibility for overspending,” has effectively controlled medical costs and reduced overall healthcare expenditures [43–45]. However, excessive cost containment may compromise medical quality to some degree [46]. Additionally, our study showed a significant decrease in the proportion of critical illness cases (16.943% vs. 18.001%) and grade IV surgeries (42.602% vs. 46.174%) after DRG implementation. This may be attributed to the fixed reimbursement structure of DRG, which provides limited payment for high-cost cases. As a result, physicians may be less inclined to manage complex and high-risk patients, potentially explaining the observed reduction in both Grade IV surgeries and critical cases. Nevertheless, no significant changes were found in hospital-acquired infection rates or mortality, suggesting that hospitals were able to maintain essential safety standards and prevent increased mortality while controlling expenditures.

To further investigate whether DRG payment influenced patient referrals patterns, we compared the logarithmic mean of total hospitalization expenditures and related medical indicators between provincial and municipal hospitals. Before DRG implementation, provincial hospitals exhibited a higher proportion of grade IV surgeries than municipal hospitals, while no significant differences were observed in the logarithmic mean of total hospitalization expenditures, proportion of critical illness cases, or mortality. After adjusting for confounding factors using linear and logistic regression analyses, the proportion of grade IV surgeries in provincial hospitals remained 1.19 times that in municipal hospitals. This may be attributed to the better medical equipment, higher professional competence of clinical teams, and greater expertise in the diagnosis, treatment, and management of complex surgeries typically found in provincial hospitals. After DRG implementation, no significant difference in mortality was observed between provincial and municipal hospitals. However, the logarithmic mean of total hospitalization expenditures, proportion of critical illness cases, and proportion of grade IV surgeries in provincial hospitals were 1.004, 1.34, and 1.44 times those in municipal hospitals, respectively. This suggests a potential shift in patient management strategies under DRG: physicians in municipal hospitals may have been more inclined to refer critically ill patients to higher-level institutions to contain costs, or even advise against further treatment for patients with poor prognosis. As a result, municipal hospitals may have admitted fewer critical illness cases and patients requiring grade IV surgeries to avoid the financial risks associated with high-cost, high-complexity care.

Given the decline in medical quality post-DRG, improving care quality has become a critical priority. **In this study, we compared data from two public hospitals before and after the** implementation **of hospital administrative interventions following DRG** adoption. **The results showed that** after the interventions, **hospital infection rates, unplanned reoperation rates, 30-day readmission rates, and patient complaint rates decreased,** while the logarithmic mean of total hospitalization expenditures increased. Similar trends were observed in regression analyses after adjusting for all confounding factors: the interventions were negatively associated with mortality, hospital infection rates, postoperative complications, and patient complaints, but positively associated with total hospitalization expenditures. These findings suggest that hospital administrative interventions can enhance medical quality, likely through optimized resource allocation, improved service efficiency, and strengthened quality control [45–48]. The incentive mechanisms embedded in such interventions may also motivate medical staff to prioritize patient safety and satisfaction, thereby reducing adverse events [48,49]. Notably, the hospital infection rate decreased significantly by 3% after the interventions, possibly due to strengthened source control measures. Reductions in unplanned re-operation rates and patient complaints further reflect the positive role of administrative interventions in improving surgical safety and patient experience. Although chi-square analysis showed no significant changes in in-hospital mortality, logistic regression adjusted for all covariates revealed a 2% reduction in mortality risk post-intervention. This discrepancy may be attributable to confounding factors, Overall, improvements in these quality indicators demonstrate the effectiveness of hospital administrative interventions. Although the logarithmic mean of total hospitalization expenditures increased after the interventions, this indirectly indicates that minimizing expenditures while maintaining quality may be impractical. Therefore, under the DRG model, hospital administrative management plays a critical role. Targeted administrative interventions—through analyzing shortcomings in clinical processes, optimizing protocols, and implementing specifical improvements—can help hospitals balance cost containment with overall medical quality [48,50], ensuring that patients receive safer and more cost-effective care under the new payment system.

## Conclusion

The implementation of the DRG payment system was associated with a reduction in average total hospitalization expenditures, thereby slowing the unreasonable rise in healthcare costs and alleviating the financial burden on both patients and insurers. Nonetheless, a primary emphasis on cost control can result in the avoidance of complex or high-risk cases, as reflected by increased patient referrals and a shift in resource allocation among hospitals. Additionally, potential reductions in diagnostic investments pose a risk to medical quality. This study confirms that hospital administrative interventions can improve medical quality, optimize resource allocation, and enhance service efficiency.

Therefore, within the DRG payment system, robust administrative intervention plays a certain role in improving medical quality, balancing expenditure control with care quality, and creating a win-win scenario for hospitals, insurers, and patients. While DRG payment system is associated with the reduction of medical expenditures, its impact on medical quality still requires ongoing attention and optimization. To ensure patients receive high-quality care under the new payment system, it is worthwhile to consider the measures such as establishing a national medical quality control center, strengthening the monitoring and evaluation of medical quality after DRG implementation, and forming internal quality improvement teams in hospitals for regular quality control assessments. These approaches will be further explored and refined in future research.

### Limitations of the study

First, this study offers a relatively broad assessment of medical quality without providing a detailed analysis across different regions or hospital tiers. Given that hospitalization expenditures and medical quality may vary significantly by geographic and institutional level, future research should incorporate these factors to enhance the validity of the findings.

Second, although stratified random sampling was used to improve data representativeness while protecting patient privacy, access to DRG payment-related data remained partially restricted. This may limit the generalizability of our results, as some patient characteristics could be underrepresented. Further validation through expanded data sources or multi-center collaborations is recommended.

Third, the observation period in this study was relatively short, which may affect the stability of the observed trends and hinder a comprehensive understanding of the long-term effects of DRG payment on medical quality. In addition, the study lacks longitudinal designs and cross-country comparisons. Future research conducted across different health systems and over extended periods would be valuable.

In summary, while this study has several limitations, they highlight meaningful directions for subsequent research. Addressing these aspects will help build a stronger theoretical and practical foundation for quality management under DRG payment systems.

## Abbreviations:

DRG: Diagnosis-Related Groups
CYN: Chinese Yuan
